# Specific cancer stem cell-therapy by albumin nanoparticles functionalized with CD44-mediated targeting

**DOI:** 10.1186/s12951-018-0424-4

**Published:** 2018-12-01

**Authors:** Yuanyuan Li, Sanjun Shi, Yue Ming, Linli Wang, Chenwen Li, Minghe Luo, Ziwei Li, Bin Li, Jianhong Chen

**Affiliations:** Department of Pharmacy, Third Affiliated Hospital & Research Institute of Surgery of Army Medical University, 10# Changjiangzhilu, Chongqing, 400042 People’s Republic of China

**Keywords:** Cancer stem cells, Hyaluronic acid, CD44, Cationic albumin, All-trans-retinoic acid

## Abstract

**Background:**

Cancer stem cells (CSCs) are highly proliferative and tumorigenic, which contributes to chemotherapy resistance and tumor occurrence. CSCs specific therapy may achieve excellent therapeutic effects, especially to the drug-resistant tumors.

**Results:**

In this study, we developed a kind of targeting nanoparticle system based on cationic albumin functionalized with hyaluronic acid (HA) to target the CD44 overexpressed CSCs. All-trans-retinoic acid (ATRA) was encapsulated in the nanoparticles with ultrahigh encapsulation efficiency (EE%) of 93% and loading content of 8.37%. TEM analysis showed the nanoparticles were spherical, uniform-sized and surrounded by a coating layer consists of HA. Four weeks of continuously measurements of size, PDI and EE% revealed the high stability of nanoparticles. Thanks to HA conjugation on the surface, the resultant nanoparticles (HA-eNPs) demonstrated high affinity and specific binding to CD44-enriched B16F10 cells. In vivo imaging revealed that HA-eNPs can targeted accumulate in tumor-bearing lung of mouse. The cytotoxicity tests illustrated that ATRA-laden HA-eNPs possessed better killing ability to B16F10 cells than free drug or normal nanoparticles in the same dose, indicating its good targeting property. Moreover, HA-eNPs/ATRA treatment decreased side population of B16F10 cells significantly in vitro. Finally, tumor growth was significantly inhibited by HA-eNPs/ATRA in lung metastasis tumor mice.

**Conclusions:**

These results demonstrate that the HA functionalized albumin nanoparticles is an efficient system for targeted delivery of antitumor drugs to eliminate the CSCs. 
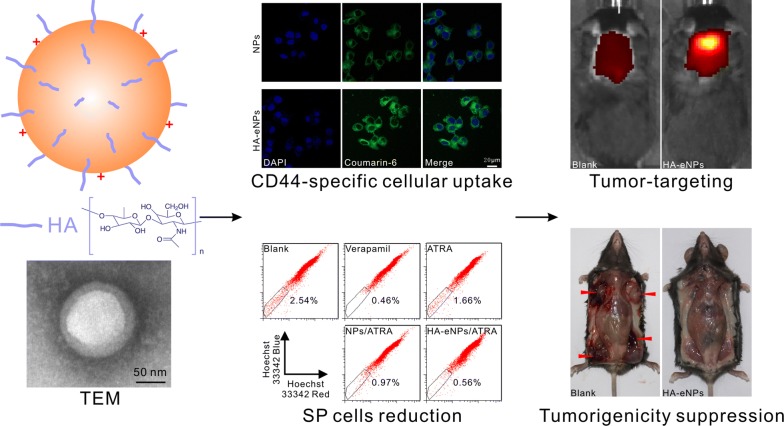

**Electronic supplementary material:**

The online version of this article (10.1186/s12951-018-0424-4) contains supplementary material, which is available to authorized users.

## Background

Despite great advances in cancer treatment, many cancer patients are still threatened by cancer drug resistance and recurrence. For instance, lung cancer remains the leading cause of cancer death in men and women [1, 2]. There is mounting evidence that cancer stem cells (CSCs) in tumor mass play crucial roles in tumor progression, chemo- and radiotherapy resistance and relapse [3–5]. Current therapeutic strategies are capable of reducing tumor bulk, but lack of specificity to kill CSCs often resulted in development of recurrence and drug resistance [6–8]. Thus, new therapeutic approaches are required to overcome the limitation of conventional treatment. Recently, CSCs became a focus for targeted therapy [9–11].

Nanoparticle systems have been extensively employed as antitumor drug carriers for enhancing their anti-tumor capacity while reducing their side effects [12–20]. They not only prevent encapsulated drugs from enzymatic degradation, but also increase drug bioavailability and accumulation at selected tissues after systemic administration [21]. Nano-drugs with certain sizes also tends to passively accumulate in targeted tumor tissues through enhanced permeation and retention (EPR) effect caused by abnormally leaky vasculature and dysfunctional lymphatic drainage system in tumor tissues [22, 23]. Therefore, the therapeutic agents are released at the tumor sites. However, EPR effect-mediated passive targeting only is insufficient to achieve excellent CSC-specific targeted therapy.

Positively charged nanoparticles have increased efficacy of drug delivery, but, they are accompanied with strong immune responses and increased removal from circulation. Recently, Lu et al. obtained cationic serum albumin through linking ethylenediamine with bovine serum albumin (eBSA) [24]. Han et al. reported eBSA-based self-assembled nanoparticles may be a promising delivery system for lung targeted therapy [25]. Nanoparticles based on the cationic serum albumin have the advantages of being nontoxic, non-immunogenic, biocompatible and biodegradable because cationization maintains protein structure and its activity [26, 27]. Additionally, modification on the surface of eBSA-based nanoparticles with tumor-targeting moieties, such as antibodies and ligands, may significantly increase the concentration of therapeutic agent on tumor site based on active targeted delivery mediated by antibody- or ligand-receptor interactions [9, 28]. Hyaluronic acid (HA), a natural biocompatible and biodegradable polysaccharide, has been used as a targeting ligand for cancer treatment due to its specific binding with CD44 receptor overexpressed on the surface of CSCs in many tumors [29]. Conjugating with HA enhances tumor targeting efficiency of normal nanoparticles. In addition, HA forms a hydrophilic layer on the surface of nanoparticles and protects the vector from opsonization, thus, prolonging systemic circulation time of HA-modified nanoparticles [30].

All-trans-retinoic acid (ATRA) is a derivative of retinoic acid, which is a naturally occurring compound known as vitamin A acid [31]. ATRA is a potent differentiating agent and involved in multiple signaling pathways related to stem cell maintenance. The anticancer efficacy of ATRA has been widely investigated in a variety of malignancies and successfully applied in clinical treatment of a stem cell malignancy, acute promyelocytic leukemia [32, 33]. The functions of ATRA rely on activating retinoic acid receptors and retinoid X receptor to regulate the transcription of genes and induce differentiation of stem cells and other biological effects [34]. However, unprotected ATRA suffers from poor solubility, instability, rapid clearance which leading to a quickly dropping in plasma concentration of ATRA and seriously dose-dependent side effects [35]. In addition, the use of ATRA is limited by low cellular uptake efficiency and lacking in tumor tissue selectivity. It is expected that encapsulated ATRA in nanoparticles may protect ATRA from degradation and increase its tumor specificity as compared with administration of free drug. Targeted delivery of ATRA into CSCs may stimulate CSCs to shift to a more differentiated status, resulting in more responsiveness to chemotherapy.

We developed new hyaluronic acid-modified nanoparticles based on eBSA. We hypothesized that HA will specifically interact with CD44 receptor resulting in selective endocytosis of the nanoparticles by CD44-enriched CSCs. The new delivery system would significantly decrease the cytotoxicity with improved differentiation initiative ability on tumor cells. The targeting effect and antitumor efficacy of the formulation were evaluated on tumor model based on CD44 enriched B16F10 cells.

## Results and discussion

### Synthesis and characterization of eBSA

Cationic BSA was obtained by modifying the surface of BSA by linking with ethylenediamine (eBSA) according with previously described method [36]. The eBSA used here has an average molecular weight (MW) of 69,614 Da analyzed by MALDI-TOF mass spectrum, indicating that each BSA molecule was on average bond 76 ethylenediamine groups (see detailed in Additional file 1: Fig. S1).

### Preparation and characterization of the HA grafted ATRA loaded cationic nanoparticles

**Fig. 1:**
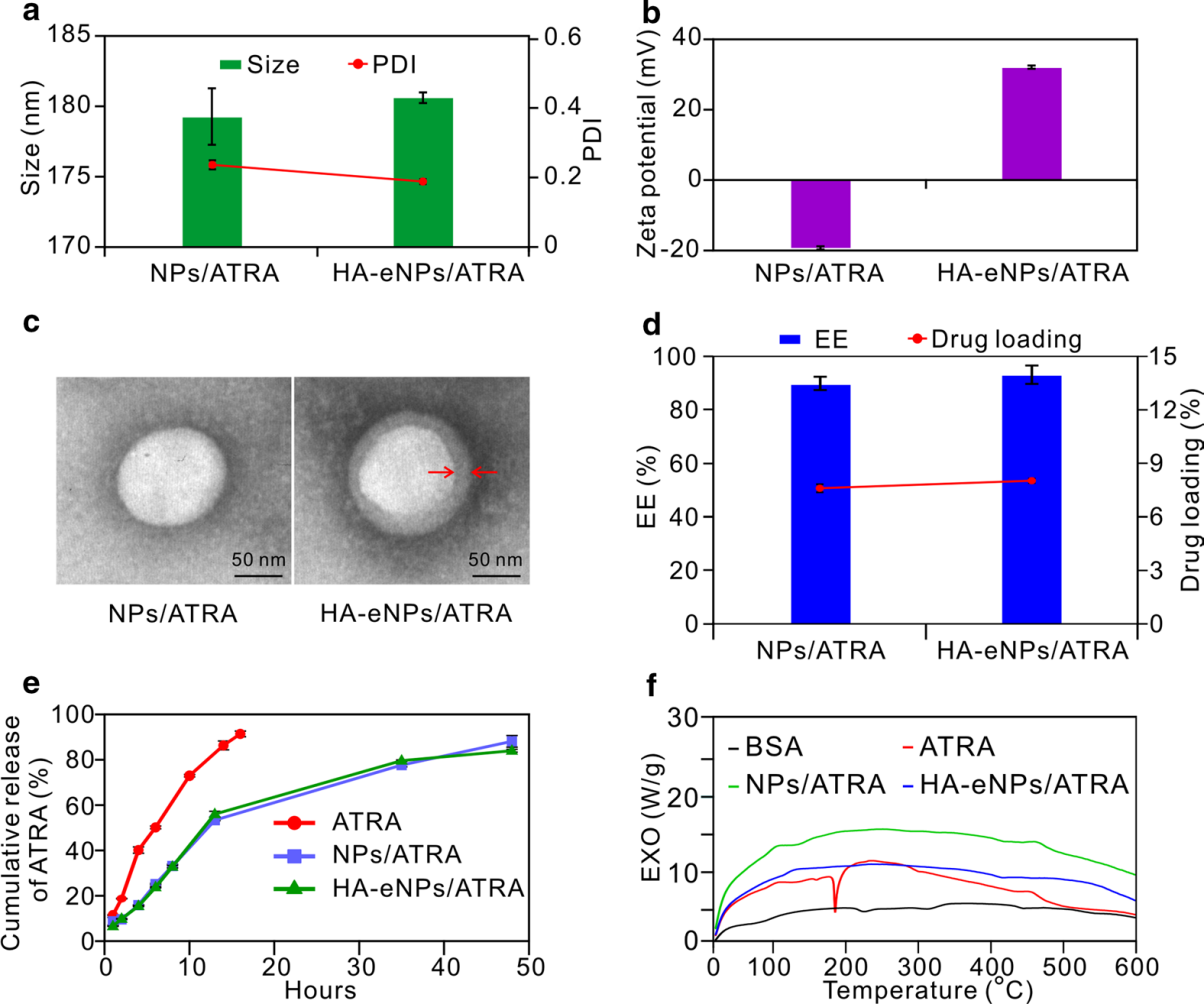
*In vitro* characteristics of NP formulations. **a**, **b** Diameter and zeta potential measurements of NP formulations. **c** Different nanoparticles visualized by transmission electron microscopy (TEM), scale bar: 50 nm (arrows indicate the coated HA layer). **d** The encapsulation efficiency and drug loading of NP formulations. Results are shown as the means ± SD (*n* = 3). **e** Release profiles of ATRA from NP formulations in PBS with 10% (*v/v*) ethanol. Results are shown as the means ± SD (*n* = 3). **f** Differential scanning calorimetry (DSC) thermograms of ATRA formulations

Albumin is an excellent material to prepare nanoparticles for drug delivery, due to its high binding capacity of drugs and good biocompatibility [27]. Nanoparticles based on cationic albumin were also designed to improve the delivery efficient into tumor site [24, 25, 37]. However, naked albumin-based nanoparticles usually are lacking in specific targeting capacity. In this research, HA was grafted onto the surface of cationic BSA-based nanoparticles, to improve the active targeting efficacy of the nanoparticles. The preparation of targeting nanoparticles system based on cationic albumin functionalized with HA (HA-eNPs) includes two steps. First step, ATRA dissolved in ethanol was added to eBSA solution and followed by homogenizing of sonication to form a crude emulsion. During the emulsion, ATRA can be encapsulated in the inside of eBSA molecule and the nanostructure (eNPs/ATRA) formed. After that, the crude emulsion was transferred into a high pressure homogenizer. During the second emulsion, the size of eNPs/ATRA should become smaller and more uniform. Second step, HA was added drop wise into eNPs/ATRA solution with stirring at room temperature. Consequently, HA was then grafted onto the surface of eNPs as a CSCs-targeting ligand in order to improve the targeting delivery efficiency. The physico-chemical properties of HA-eNPs were investigated. Dynamic light scattering (DLS) results showed that ATRA-loaded normal BSA nanoparticles (NPs/ATRA) and HA modified cationic nanoparticles (HA-eNPs/ATRA) have an average size of 179.26 ± 1.98 nm, 180.63 ± 0.38 nm, and a polydispersity index (PDI) of 0.227 ± 0.013, 0.180 ± 0.007, respectively (Fig. 1a). The zeta potentials were − 19.3 ± 0.62 mV and 32.1 ± 0.42 mV, respectively (Fig. 1b). TEM analysis revealed that the nanoparticles are spherical and surrounded by a grey ring, suggesting that HA formed a coating layer on the surface of the eNPs (Fig. 1c, right). The quantity of HA was estimated to be on average 72.9 μg of HA per milligram HA-eNPs/ATRA (Additional file 1: Fig. S2). Further studies revealed that ATRA was well incorporated into nanoparticles with encapsulation efficiencies of 89.8% for NPs/ATRA, 93.0% for HA-eNPs/ATRA (Fig. 1d). So, the presence of HA at the surface of nanoparticles did not diminish the encapsulation of ATRA. The drug loading capacity of HA-eNPs/ATRA was 8.37%, according to the percentage of ATRA in HA-eNPs/ATRA. These results suggest that HA-eNPs/ATRA were successfully prepared.

The in vitro release studies of the HA-eNPs were performed using a dialysis technique in PBS at pH 7.4, alongside with free ATRA. All samples were analyzed by high performance liquid chromatography (HPLC). The release profiles are shown in Fig. 1e. The profile of free ATRA showed 40% drug-release at the sampling time of 4 h and more than 80% by 12 h. However, it was less than 60% for NPs/ATRA and HA-eNPs/ATRA. NP formulations exhibited sustained ATRA release up to 48 h, reaching 88.1% for NPs and 84.0% for HA-eNPs (Fig. 1e).

To investigate the physical state of the drug incorporated in the NP formulations, DSC analysis was performed to determine the thermal properties of the nanoparticles. As shown in Fig. 1f, ATRA exhibited a sharp endothermic peak at 185 °C, which indicates that free ATRA are in the crystal state. After incorporation into NPs, all characteristic peaks of ATRA disappeared. Thus, the majority of ATRA encapsulated in the NPs was in an amorphous or disordered crystalline phase dispersed in BSA matrix [38].

### Stability analysis of HA-eNPs

**Fig. 2:**
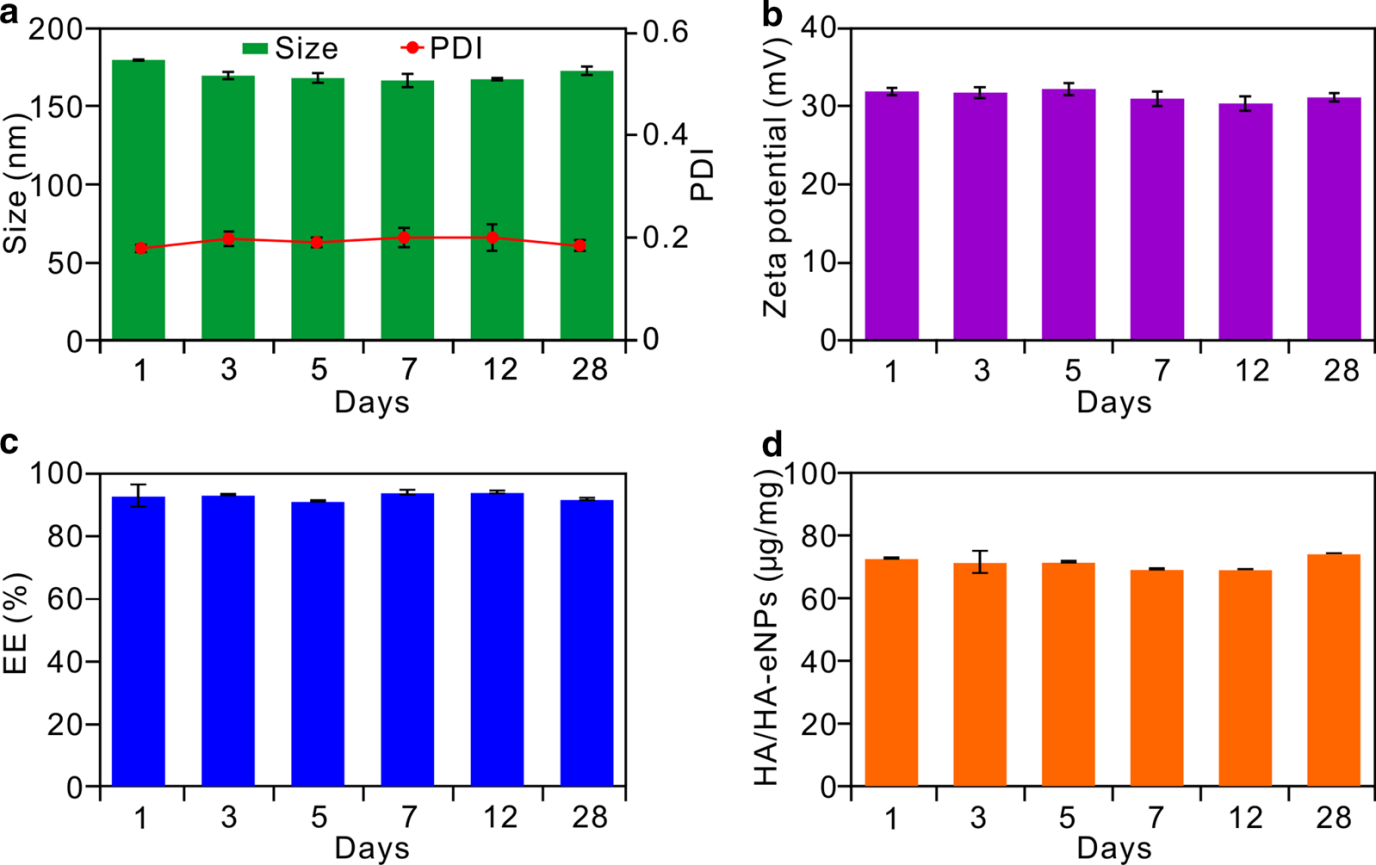
Stabilities evaluation of ATRA loaded HA-eNPs. Continually determinations of **a** the diameter, **b** the zeta potential, **c** the encapsulation efficiency, and **d** HA density of HA-eNPs within 4 weeks, respectively. Results are shown as the means ± SD (*n* = 3)

Good pharmaceutical preparations should have good stability for a certain period of time, so that they can be easily used in clinic. Therefore, the physical stability of HA-eNPs/ATRA was assessed in a period of 4 weeks storage at 2–8 °C in the refrigerator. As shown in Fig. 2a, b, no significant changes on sizes and zeta potentials were found within 4 weeks. In the end of evaluation, the diameter was 173.80 nm with a PDI of 0.184 and the zeta potential was 31.2 mV. Moreover, the good stability of HA-eNPs was confirmed by the high stable encapsulated efficiency of ATRA (Fig. 2c). In addition, the stability of HA conjugation was also assessed by continuous detecting density of HA on the surface of HA-eNPs. As shown in Fig. 2d, there was no significant change on density of ligand observed, showing 74.5 μg per milligram HA-eNPs/ATRA on day 28. These results demonstrated that the nanoparticles can be preserved for 4 weeks before it is used.

### Cellular uptake of HA-eNPs

**Fig. 3:**
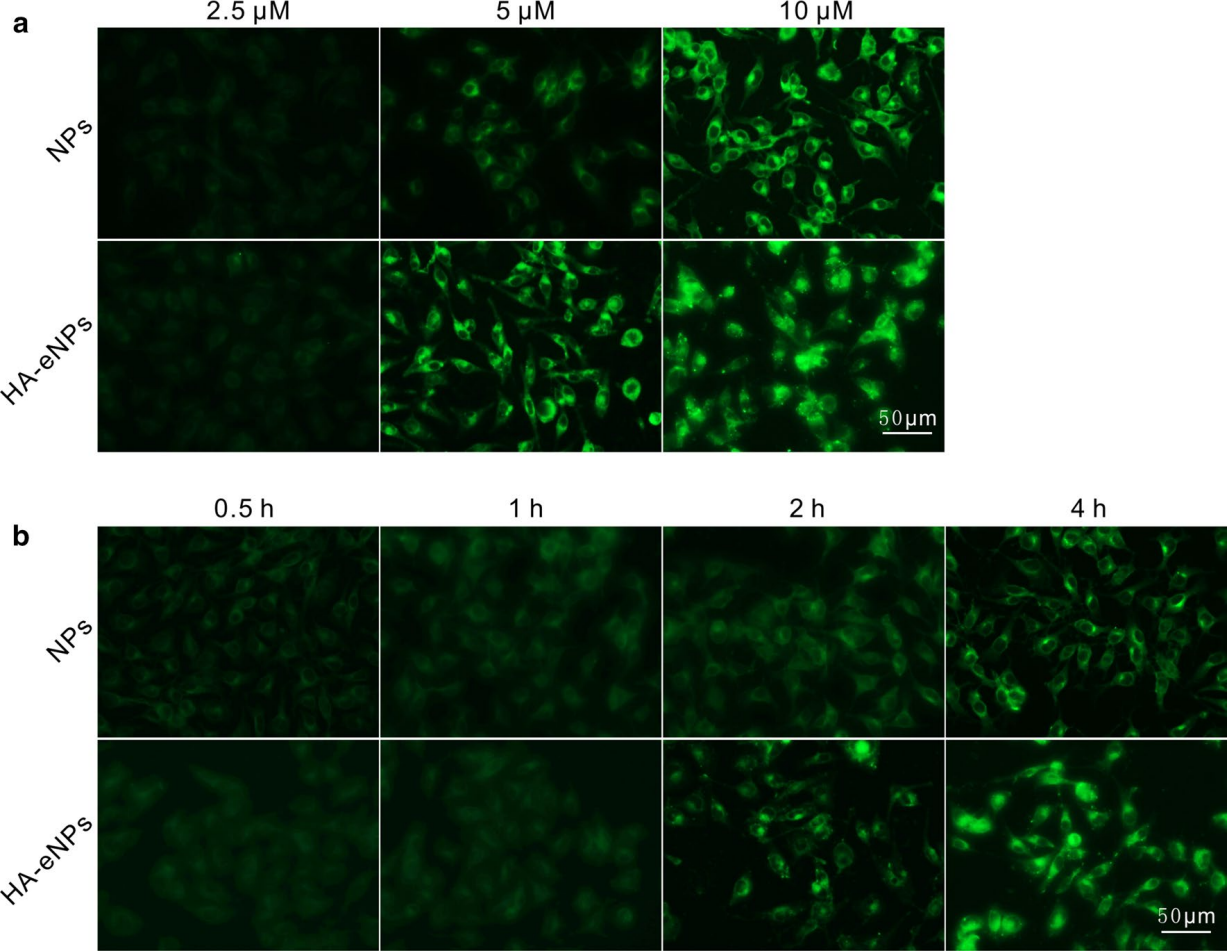
Fluorescence analysis of uptake of HA-eNPs/ATRA in B16F10 cells. **a** Fluorescence images of uptake of coumarin-6-labeled NPs at different ATRA concentrations (4 h). **b** Fluorescence images of uptake of coumarin-6-labeled NPs at different time points (5 μM ATRA). Fluorescence signals were observed by fluorescence microscopy. Scale bar represents 50 μm

The cellular uptake and distributions of NP formulations were determined by fluorescence microscopy and flow cytometry. To facilitate the detection, coumarin-6 was incorporated into NPs as a fluorescent signal. B16F10 cells seeded in 6-well culture plate were incubated with NP formulations at different concentrations and times. As shown in Fig. 3a, b, both two NP formulations were uptaken in a dose- and time-dependent manner. In addition, cellular uptake of HA-eNPs was significantly stronger than that of normal NPs. The result suggested that HA grafting onto nanoparticles facilitated cellular uptake of anticancer drug.

**Fig. 4:**
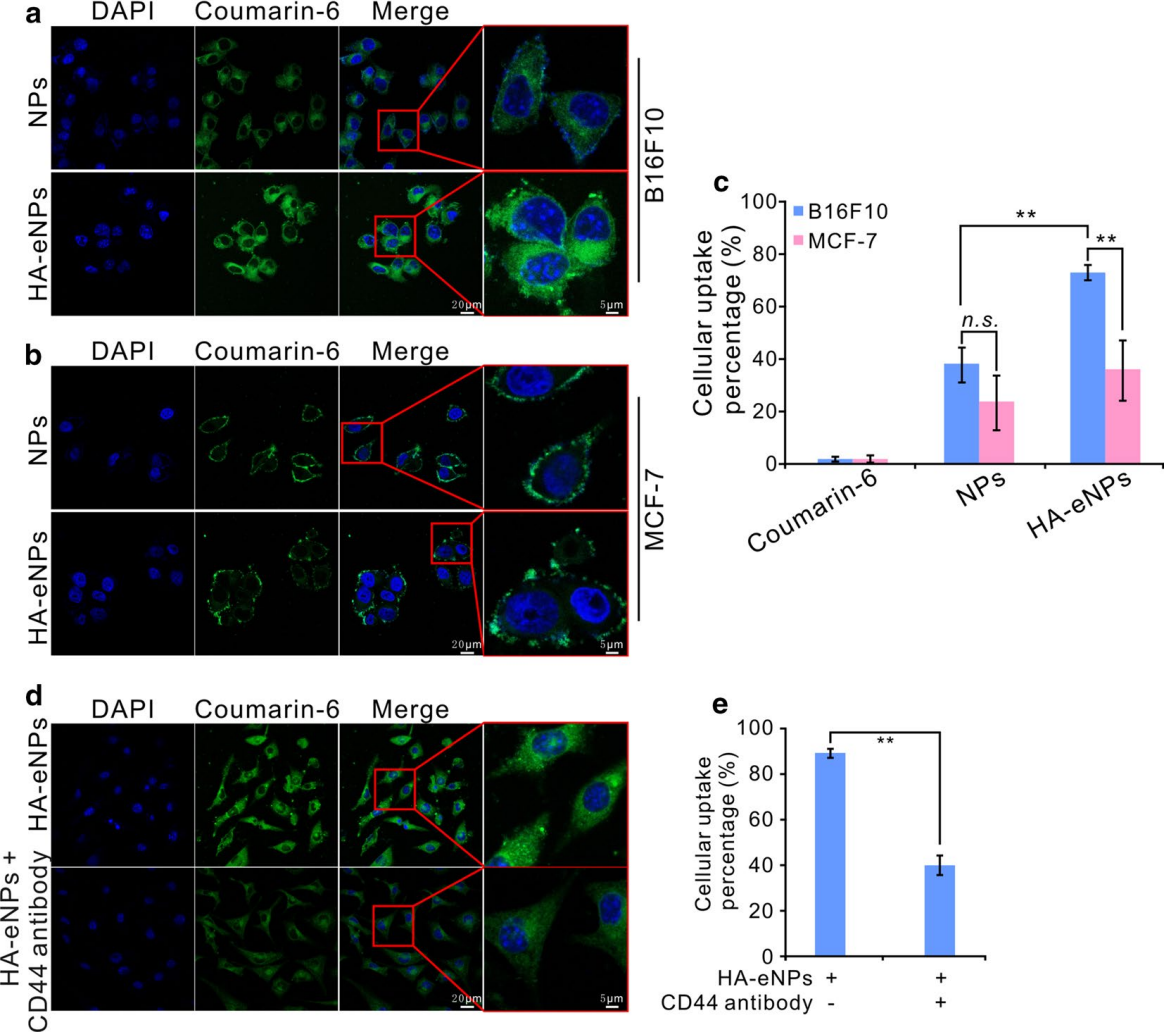
In vitro evaluation of detecting the targeting of HA-eNPs to CD44. **a**, **b** Coumarin-6 loaded HA-eNPs were incubated with CD44-enriched B16F10 cells or CD44-low expressing MCF-7 cells, respectively. Fluorescence signals were observed by confocal microscopy. **c** Flow cytometry analysis showing the cellular uptake percentage of the B16F10 and MCF-7 cells after incubation with free coumarin-6, coumarin-6 loaded NPs, or HA-eNPs. Data are shown as the means ± SD, ^**^P < 0.01, *ns* not significant (*n* = 3). **d**, **e** B16F10 cells were preincubated with anti-CD44 antibody and followed by incubating with HA-eNPs for 4 h. Fluorescence signals were observed by confocal microscopy and flow cytometry. Data are shown as the means ± SD, ^**^P < 0.01 (*n* = 3)

To evaluate the efficiency of HA/CD44 mediated specific cellular uptake, the CD44-enriched B16F10 cells or CD44 low-expressing MCF-7 cells were co-incubated with NPs or HA-eNPs for 4 h in serum-free medium [39]. Then, the fluorescence signals of NP formulations were examined by confocal microscopy and flow cytometry. As shown in Fig. 4, the fluorescence intensity of HA-eNPs cell group was significant higher than that of normal NPs cell group in CD44-enriched B16F10 cells (Fig. 4a). Whereas, no significant increased fluorescence signal was observed in HA-eNPs group comparing with normal NPs group in CD44 low-expressing MCF-7 cells (Fig. 4b, Additional file 1: Fig. S4). Then, the cellular uptakes of ATRA in the two tested cancer cells were quantitatively evaluated via flow cytometry. As shown in Fig. 4c, HA-eNPs group showed significant higher cellular uptake percentage (73.3%) than that of NPs group (38.1%, *P* < 0.01) in B16F10 cells, respectively. However, only weak increase of cellular uptake percentage in HA-eNPs group was observed (23.7%) compared with that of NPs group (36.0%, *P* > 0.05) in MCF-7 cells. To further investigate the endocytosis mechanism of the nanoparticles, anti-CD44 antibody blocking experiments were performed. Figure 4d, e showed that the fluorescence signals in the HA-eNPs treated cells were significantly reduced after blocking by anti-CD44 antibody (*P *< 0.01). This phenomenon can attributed to the saturation of the receptor caused by CD44-antibody reaction prevented the binding of HA-coated NPs to its receptor. Those results revealed that the uptake of HA-eNPs by cancer cells is related to their CD44 level of expression. These results confirm the interest of nanoparticle functionalized with HA in the aim of CD44 over expressing cancer therapy.

### HA-eNPs exhibited potent cell growth inhibition and apoptosis induction effects on B16F10 cells

**Fig. 5:**
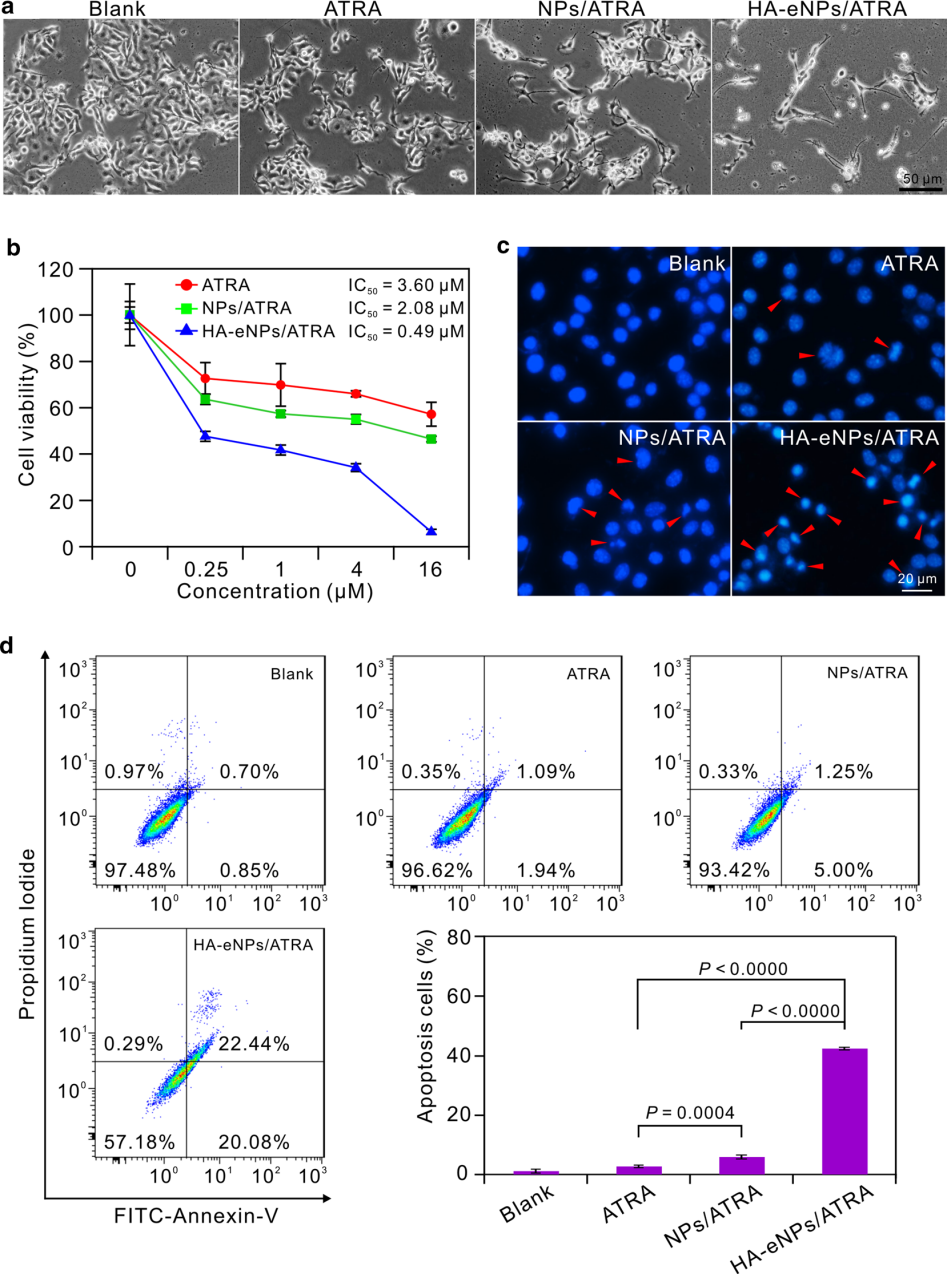
In vitro antitumor effects of NP formulations on the B16F10 cells. **a** Morphological changes of B16F10 cells after treatment with various ATRA formulations. Scale bar represents 50 μm. **b** MTT viability assay for B16F10 cells after treatment with various ATRA formulations. Results are shown as the means ± SD (*n* = 3). **c** Apoptosis of B16F10 cells induced by various ATRA formulations (5 μM) (arrows indicate cell apoptosis). Scale bar represents 20 μm. **d** Flow cytometry analysis of B16F10 cell apoptosis induced by ATRA formulations. Results are shown as the means ± SD (*n* = 3)

To investigate the possibility of utilizing the HA-eNPs for drug delivery, we tested the killing ability of the HA-eNPs on cancer cells. The cytotoxicity of HA-eNPs was evaluated using the MTT assay with the CD44-enriched B16F10 cells. The ATRA, NPs/ATRA, and HA-eNPs/ATRA containing equivalent concentrations of ATRA were used. As shown in Fig. 5a, untreated B16F10 cells were bright and crowded with a fusiformis appearance, whereas treated cells showed morphological changes including decreased density and increased floating cells. Interestingly, growth of the cells treated with HA-eNPs/ATRA was great inhibited and the remaining few cells became rounded, irregular or shrunken and detached from each other. Furthermore, cell viability decreased progressively with increasing ATRA concentrations. We noted that HA-eNPs/ATRA exhibited the most potent cell growth inhibitory effect than free ATRA and NPs/ATRA. When ATRA concentration of HA-eNPs increased to 16 μM, there was only 7% cells viable left, while 57%, 46% for free ATRA and NPs/ATRA treated cell, respectively (Fig. 5b). Incorporation in HA-eNPs greatly reduced IC_50_ of ATRA from 3.60 to 0.49 μM. However, HA-eNPs did not exhibit much increasing of cytotoxicity on MCF-7 cells comparing with NPs (see Additional file 1: Fig. S4). These results suggest that the enhanced cytotoxicity was likely resulted from improved uptake of ATRA by HA-CD44 interations, with increased intracellular concentration of ATRA.

To further investigate the influence of ATAR formulations on cell apoptosis, cell nuclei DAPI staining and annexin V-FITC and propidium idodide (PI) staining were performed. As shown in Fig. 5c, the nuclei of untreated cells displayed bright blue fluorescence and homogeneous chromatin. However, a number of cells showed extreme chromatin condensation and nuclear fragmentation in HA-eNPs/ATRA treated group. Similarly, flow cytometry showed that all treated cell groups had increased fraction of apoptotic cells (annexin V^+^/PI^+^ and annexin V^+^/PI^−^) compared with control cell (Fig. 5d). Notably, HA-eNPs/ATRA induced the greatest increase in annexin V^+^/PI^+^ cells (22.44%) and annexin V^+^/PI^−^ cells (20.08%). These observations indicate that HA-eNPs enhanced the apoptosis inducing effect of ATRA on CD44-enriched B16F10 cells. It is probably due to the HA modification, which facilitated the uptake of ATRA by the CD44-enriched B16F10 cells.

### HA-eNPs altered the distribution of ATRA in mice

**Fig. 6:**
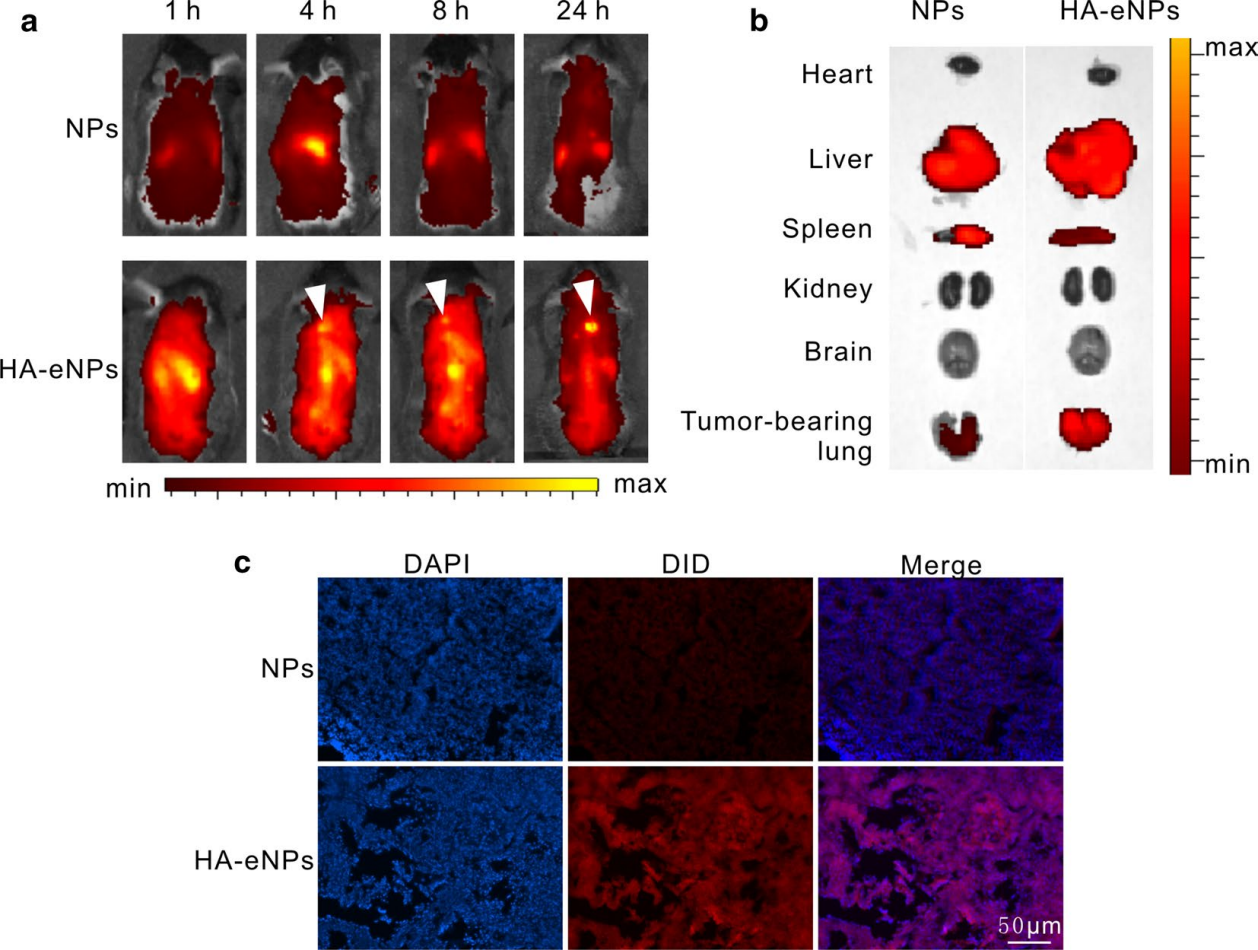
Fluorescence imaging of ATRA-loaded NP formulations in mice. **a** Time-dependent intensity images of fluorescence distribution in mice (arrows indicate accumulation of HA-eNPs in tumor-bearing lungs). **b** Fluorescence images of major organs at 8 h post-injection of NP formulations in mice. **c** Fluorescence microscopy images of tumor-bearing lung tissue section. Scale bar represents 50 μm

In vivo fluorescence imaging analysis was performed to investigate the biodistribution and tumor targeting properties of HA-eNPs/ATRA. DiD was incorporated into NPs as the fluorescence signals. DiD signals were monitored after intravenous administration of DiD-labeled NP formulations into the lung metastasis tumor-bearing mice. Figure 6a showed real-time images of NPs in the tumor-bearing mice, in which the whole bodies of live mice were monitored over the course of 24 h. Considerable fluorescence was detected and gradually decreased in the whole body, resulting from circulation of NPs in the bloodstream. As well know, CD44 plays a critical role in regulating CSCs stemness properties and has been identified as a typical CSCs surface marker for enriching or targeting CSCs in various types of cancer [40]. Meanwhile, CD44 is a broadly distributed transmembrane glycoprotein in our body, the smallest standard isoform (CD44s) is found in most cells, although its variants (CD44v1–v10) primarily overexpress on tumor cells [41, 42]. Consequently, parts of HA-eNPs could bind with CD44 expressing on other tissues besides metastatic B16F10 tumor cells. However, DiD signals clearly visible in the whole mouse body except for tumor-bearing lungs (as shown in Fig. 6a), were mainly resulted from DiD flowing in the circulation system. Interestingly, HA-eNPs exhibited the strongest fluorescent signal in the tumor-bearing lung compared with NPs and eNPs (Fig. 6a and Additional file 1: Fig. S5) mouse groups, which indicating that more HA-eNPs accumulated in the tumor-bearing lungs. In HA-eNPs group, DiD signal in the lung reached maximum at 4 h post-injection and remained strong until 24 h post-injection. This high tumor targetability of HA-eNPs might be due to a combination of an EPR effect, as well as receptor-mediated endocytosis of HA-eNPs. In order to confirm the lung tumor targeting effect of HA-eNPs, major organs from euthanized mice were imaged under 8 h post-injection (Fig. 6b). As expected, strong DiD signal in tumor-bearing lungs was observed in HA-eNPs mouse group, whereas DiD in NPs groups was very weak. Furthermore, fluorescence microscopy images of lung tissue section from mice 8 h post-injection with DiD-labeled NPs confirmed that HA-eNPs accumulated in the tumor-bearing lung tissue (Fig. 6c). It was validated that the introduction of HA offered the nanoparticles an excellent tumor targeting efficacy, leading to a higher efficient cancer treatment.

### HA-eNPs/ATRA suppressed the tumorigenicity of CD44-enriched B16F10 cells

**Fig. 7:**
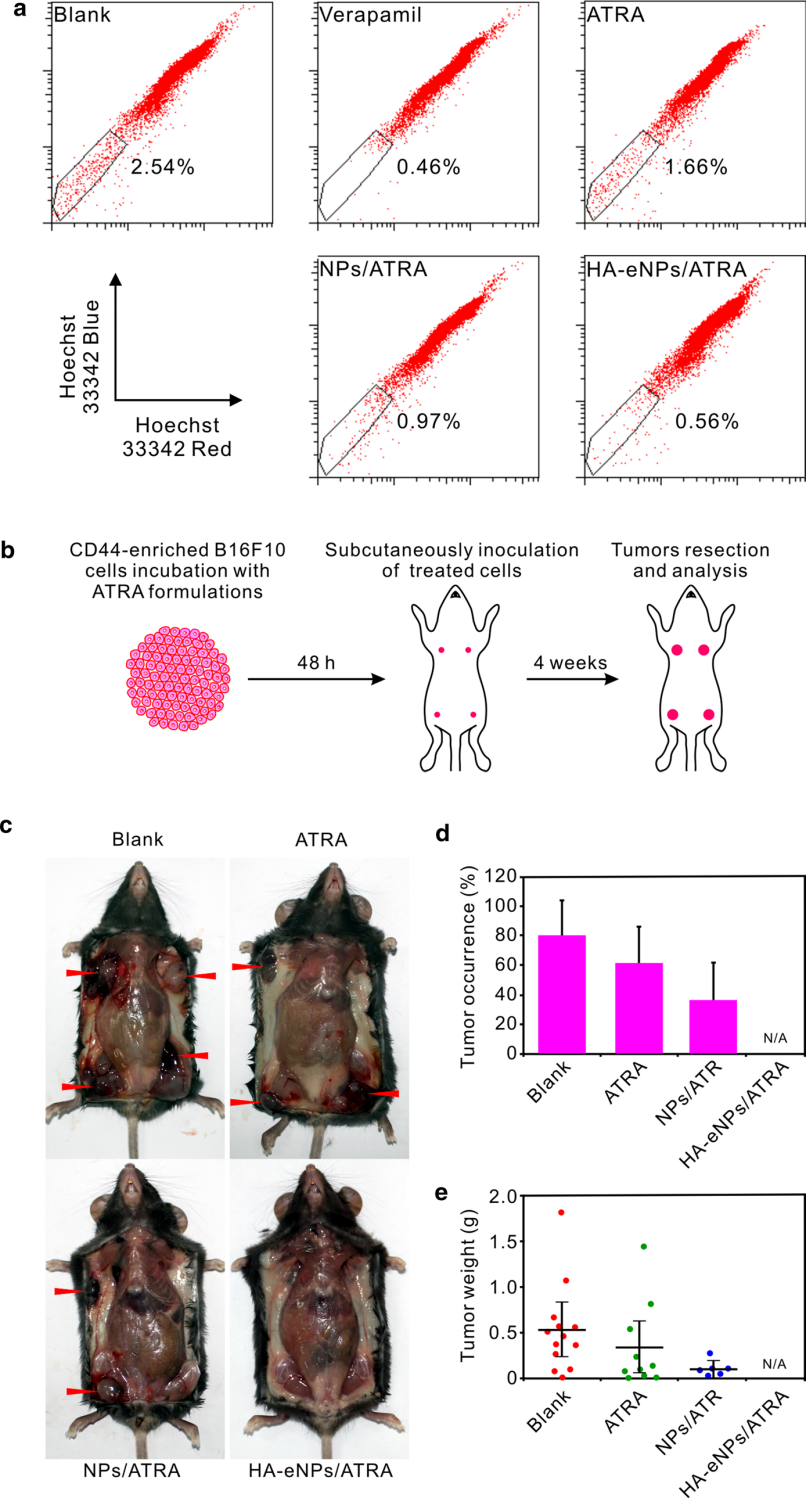
HA-eNPs/ATRA decreased the tumorigenicity of CD44-enriched B16F10 cells. **a** Side population (SP) changes in B16F10 cells after treatment with various ATRA formulations. **b** A diagram of the tumorigenicity assessment. In the experiment, four flanks of each mouse were injected with the same treated or untreated B16F10 cells. **c** Representative photo of tumors 24 days after subcutaneous implantations of ATRA formulations treated or untreated B16F10 cells (arrows indicate tumor nodules). **d**, **e** Tumor occurrences and weights measured 24 day after implantation. Results are shown as the means ± SD (*n* = 16)

Side population cells (SP cells), with some stem cell properties, have been identified in cancers where they show increased capacity of self-renewal and tumorigenicity in vivo [43, 44]. To evaluate the influence of HA-eNPs/ATRA on the tumorigenicity of CD44-enriched B16F10 cells, SP cells analysis and in vivo xenograft experiments were performed. As shown in Fig. 7a, after 48 h treatment, ATRA or its NP formulations reduced the proportion of SP cells compared with untreated B16F10 cells. HA-eNPs/ATRA most potently decreased the proportion of SP cells (from 2.54 to 0.56%), indicating that HA modification facilitated specific delivery of ATRA into CD44-enriched B16F10 cells.

Cancer stem cells are more proliferative and tumorigenic, injection of few CSCs will give rise to tumor development. Therefore, the influence of HA-eNPs on tumorigenicity was assessed by subcutaneously inoculating ATRA formulations treated and untreated B16F10 cells into the flanks of C57BL/6 mice (Fig. 7b). After 24 days, as shown in Fig. 7c–e, the untreated cells gave rise to more tumors than the treated cells, with an average occurrence of 81.25% and tumor weight of 0.54 g. ATRA and NPs/ATRA treated cells generated less tumors, with average occurrence of 62.5% and 37.5%, average tumor weight of 0.35 g and 0.11 g, respectively. However, no tumor formed in all mice injected with HA-eNPs/ATRA treated B16F10 cells. Similar trends were also observed in another xenograft experiment, in which four flank sites of each mouse were injected with different treated or untreated B16F10 cells, respectively (Additional file 1: Fig. S6). HA-eNPs/ATRA exhibited the best tumorigenicity suppression effect among all of the formulations, probably due to efficient stem-like cell targeting delivery of ATRA resulted from HA/CD44 mediated endocytosis.

### Inhibition of tumor growth

**Fig. 8:**
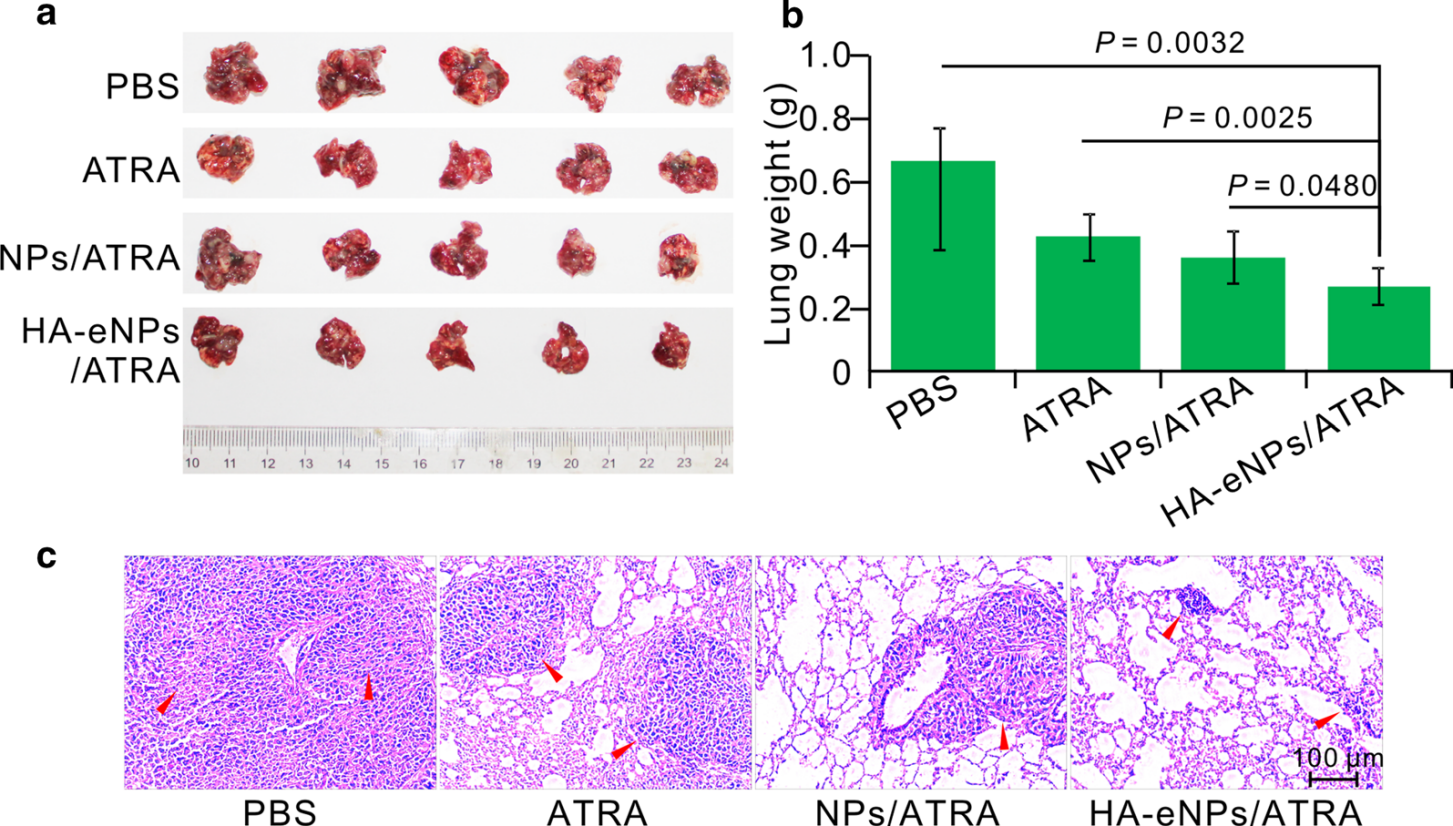
HA-eNPs/ATRA enhanced the inhibition of tumor growth in the in situ lung metastasis tumor-bearing mice. **a** Images of the B16F10-bearing lungs on day 24 after five consecutive *i.v.* injections of NP formulations (n = 5). **b** Antitumor effects of various treatments evaluated according to B16F10-bearing lung weight (n = 5). **c** Histological staining of the B16F10-bearing lungs after various treatments (arrows indicate tumor nodules, n = 5)

In this study, in situ lung metastasis tumor-bearing mice were employed to assess the inhibitory effect of HA-eNPs/ATRA on tumor growth. Their tumor morphology, weights, and histology were analyzed. As shown in Fig. 8a, tumor nodules covered nearly the entire surface of the excised lungs in the untreated tumor-bearing mice. The tumor nodules in the lungs of ATRA-, NPs/ATRA-, and HA-eNPs/ATRA-treated groups decreased compared with that of PBS-treated group. The average weights of the tumor-bearing lungs were analyzed and are shown in Fig. 8b. As expected, the B16F10-bearing lung weights of ATRA-containing formulations-treated groups significant decreased compared with the PBS groups. More importantly, the HA-eNPs/ATRA exhibited the highest inhibitory effect compared with the NPs/ATRA group (*P *< 0.05) and the free drug group (*P *<0.01). Furthermore, the H&E staining analysis revealed that, tissue specimens from the HA-eNPs/ATRA treated mice shown dramatically lower densities of tumor cells than those of other groups (Fig. 8c). Based on these results, we can conclude that the HA-eNPs/ATRA exhibited the best antitumor effect due to the targeting to stem-like cells, which relies on HA/CD44 interactions.

### Preliminary safety evaluation

**Fig. 9:**
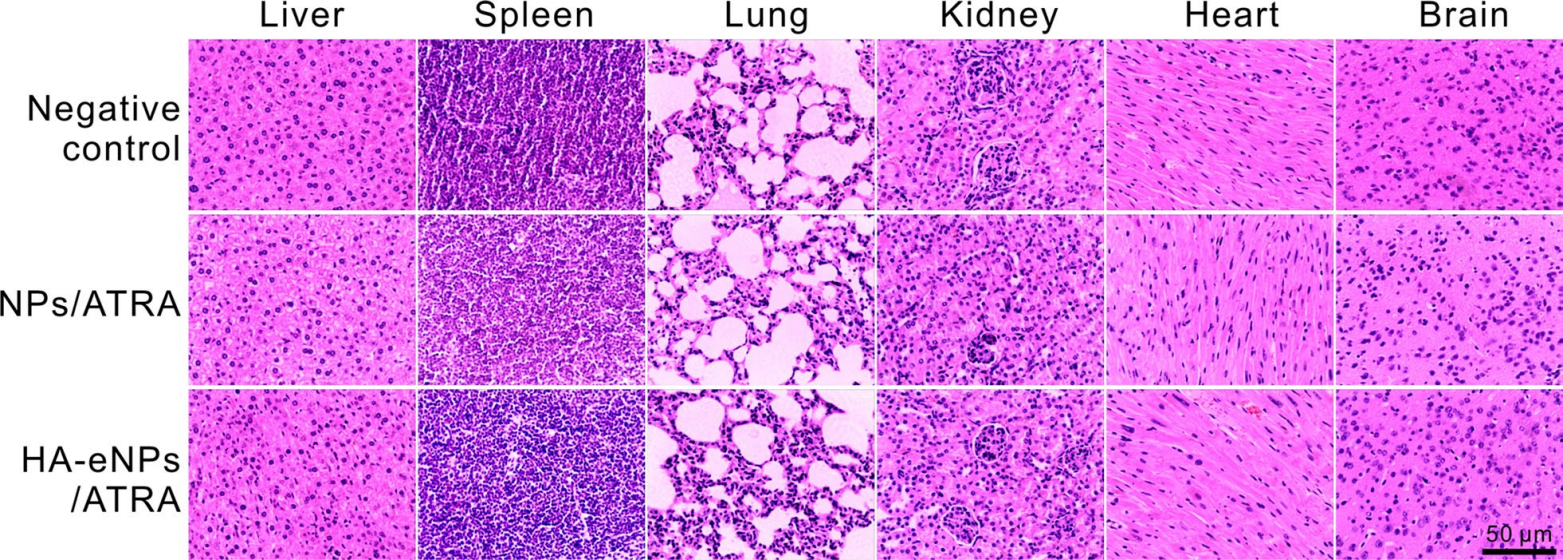
Safety evaluation of ATRA-loaded NP formulations. Microscopic images of hematoxylin and eosin (H&E) staining of organs (liver, spleen, lung, kidney, heart and brain) from mice treated with ATRA-loaded NP formulations. No abnormality was observed. Scale bar represents 50 μm

To evaluate in vivo toxicity of NP formulations, hematoxylin and eosin (H&E) staining of organs from treated mice was performed (Fig. 9). Histopathologic examination showed no abnormalities in those organs from treated mice compared with negative control mice, suggesting that NP formulations are biocompatible. These results indicate that encapsulation of ATRA into NPs reduces the toxicity of drugs, and HA-grafted NPs are excellent carriers for therapeutics, especially for targeting CD44-overexpressing cancers.

## Conclusion

The study herein developed a kind of cationic albumin nanoparticle system functionalized with hyaluronic acid to target the CD44 overexpressed CSCs. The delivery system had high entrapment efficiency as well as good stability. The in vitro drug release profile revealed that the HA-eNPs displayed a sustained and prolonged release. The fluorescence images demonstrated the efficient and specific cellular uptake of HA-eNPs. In vivo biodistribution illustrated that HA-eNPs can selective accumulate in tumor-bearing lung of mouse. The MTT and apoptosis experiments indicated that HA-eNPs/ATRA showed a much higher cytotoxicity than the free drug. Finally, tumor growth was significantly inhibited by HA-eNPs/ATRA in lung metastasis tumor mice. This work demonstrate that the HA functionalized cationic albumin nanoparticles is an efficient system for targeted delivery of antitumor drugs to eliminate the CSCs.

## Methods

### Materials

Bovine serum albumin (BSA MW 66430 Da) was purchased from Sinopharm Chemical Reagent Co., Ltd (Beijing, China). Hyaluronic acid (HA, ~ 10,000 Da polymers) was obtained from Bloomage Freda Biopharm Co., Ltd. (Shandong, China). 1-Ethyl-3-(3-dimethylaminopropyl)carbodiimide hydrochloride (EDCI·HCl) was purchased from Aladdin Chemistry Co., Ltd (Shanghai, China). Coumarin-6 and DAPI were bought from Sigma-Aldrich (St. Louis, MO). All-trans-retinoic acid (ATRA) was purchased from Saen Chemical Technology Co., Ltd (Shanghai, China). RPMI-1640 cell culture medium was supplied by Gibco (Life Technologies, Switzerland). 3-(4, 5-methylthiazol-2-yl)-2, 5-diphenyltetrazolium bromide (MTT) was obtained from Amresco (Solon, Ohio, USA).

### Cell lines and animals

Murine melanoma cell line B16F10 was cultured in RPMI-1640 medium with 10% fetal bovine serum, 1% penicillin/streptomycin. MCF-7 breast cancer cells were purchased from the American Type Culture Collection (ATCC, Manassas, VA), which were cultured in Dulbecco’s Modified Eagle’s Medium (DMEM) with 10% fetal bovine serum, 1% penicillin/streptomycin. Female C57BL/6 mice (6–8 weeks) were obtained from the Laboratory Animal Center of Army Military Medical University (Chongqing, China). All animal experiments were approved by the Institutional Animal Care and Ethics Committee of Army Military Medical University.

### Synthesis and characterization of ethylenediamine conjugated bovine serum albumin (eBSA)

First, 500 mg BSA (dissolved in 5.0 mL distilled water), and 150 mg EDCI·HCl were added to 40 mL 1.4 M ethylenediamine solution (pH = 4.75) and stirred at room temperature for 2 h. Then, 400 μL 4 M acetate buffer (pH = 4.75) was added to terminate the reaction. The product was purified by bag filter (molecular cut off: 7 kDa) to remove free ethylenediamine and EDCI. Water was removed by freeze-drying. The product was analyzed by MALDI-TOF–MS (Shimadzu MALDI-7090).

### Preparation and physiochemical characteristics of the HA grafted ATRA loaded cationic nanoparticles

First, ATRA-loaded BSA nanoparticles (NPs/ATRA) were prepared according to description previously [45]. Briefly, 4.5 mg of ATRA was dissolved in 1 mL ethanol. The solution was added to 10 mL of BSA solution (0.5% *w/v* in distilled water). The mixture was homogenized by sonication for 15 min in order to form a crude emulsion, and then transferred into a high pressure homogenizer (ATS Engineering Inc., USA). The emulsification was performed at 15,000 psi for 20 cycles. The resulting system was transferred into a rotary evaporator to remove ethanol. Secondly, ATRA-loaded eBSA nanoparticles (eNPs/ATRA) were prepared according to the description above. At last, HA was added into the eNPs/ATRA solution with an HA/eBSA ratio of 1:5 (w/w) and stirred for 20 min at room temperature to prepare the targeting delivery system (HA-eNPs/ATRA).

The size distribution and zeta potentials were measured by photon correlation spectroscopy (PCS) (Malvern zetasizer Nano ZS90, UK). Transmission electron microscope (TEM) was adopted to study the morphologies of the NP formulations which were disposited onto a copper grid and then stained with phosphotungstic acid (1%) for 10 s before observation using TEM instrument (TEM-1400 plus, JEOL).

To determine the entrapment efficiency, nanoparticles were separated from the dispersion by centrifugation at 15,000*g* for 90 min. The supernatant was analyzed for free ATRA by high-performance liquid chromatography (HPLC) (Kromasil ODS-1 C18 column (150 × 4.6 mm, 5 μm); mobile phase: CH_3_COONH_4_ (0.1 M): CH_3_OH = 12:88 (v/v); flow rate: 1 mL/min; wavelength: 348 nm). Encapsulation efficiency (EE %) and drug loading yield (DL) of NPs were calculated by the following equations.

1 $$ {\text{Encapsulation efficiency}}\%  = \frac{{{\text{weight of the feeding drug}} - {\text{weight of free drug}}}}{\text{weight of the feeding drug}} \times 100 $$

2 $$ {\text{Drug loading yield}}\%   = \frac{\text{weight of encapsulated drug in NPs}}{\text{total weight of NPs}} \times 1 0 0 $$

ATRA release from NPs was monitored by dialysis using a bag filter (molecular cut off: 7 kDa, Millipore, USA), against 50 mL PBS with 10% (v/v) ethanol (pH 7.4). At designated time intervals, aliquots were removed from the dialysate and replaced by an equal volume of PBS/ethanol buffer. The amount of ATRA in the dialysate was determined by HPLC (Agilent Technologies, Santa Clara, CA).

### Differential scanning calorimetry (DSC)

DSC (DSC 200F3, NETZSCH) analysis was carried out for free BSA and ATRA, freeze dried ATRA-loaded NPs and HA-eNPs. All samples were weighted and sealed in an aluminum cell and then heated from 25 °C to 350 °C at a rate of 10 °C/min under a nitrogen atmosphere.

### Stability of nanoparticles

The physical stability of ATRA loaded HA-eNPs were evaluated after storage at 2–8 °C for up to 4 weeks. At each time point, an aliquot of ATRA loaded HA-eNPs dispersion was collected to measure the mean particle size, polydispersity index (PDI), and surface potential by photon correlation spectroscopy (PCS) (Malvern zetasizer Nano ZS90, UK), EE% by HPLC (Agilent 1290, USA), and HA density by UV–vis spectrophotometer (Persee, China). The measurements were performed in triplicate.

### Cell uptake assay

The cellular uptake and distribution of NP formulations were analyzed by fluorescence microscopy and flow cytometry. The CD44-enriched B16F10 cells were seeded in a 6-well culture plate 1 day before treatment with NPs. Coumarin-6 was incorporated into the NPs to obtain fluorescence-labeled formulations. CD44-low expressing MCF-7 cells were employed as the negative control to evaluate the HA mediated cellular uptake. After incubation with the fluorescence-labeled formulations, including NPs/ATRA and HA-eNPs/ATRA without fetal bovine serum at 37 °C, the cells were washed by PBS and analyzed by fluorescent microscopy (EVOS FL, Life Technologies, USA), confocal microscopy (LSM 800, Zeiss, Germany), and a flow cytometer (Novocyte, ACEA, USA). For the blocking experiments, B16F10 cells were first incubated with anti-CD44 antibody (20 μg/mL, ab112178, abcam) for 1 h. After removing the antibody, the cells were incubated with coumarin-6 labeled HA-eNPs for 4 h. Cells were then treated as described above.

### Assessment of cell growth inhibition and apoptosis induction

The proliferation of drug-treated CD44-enriched B16F10 cells was determined by MTT assays. Briefly, the cells were seeded in 96-well culture plates and allowed to attach. On the next day, the cells were exposed to ATRA or NP formulations for 48 h at a series of ATRA concentrations and followed by addition of 20 μL MTT (5 mg/mL) to each well. The cells were continually incubated in total darkness for 4 h. Then, the supernatant was replaced by 150 μL DMSO in order to ensure the formazan crystals dissolved. Cell viabilities were determined by measuring the absorbance at 570 nm using a Wellscan MK3 microplate reader (Thermo, USA).

Cell apoptosis was assessed by 4′6-diamidino-2-phenylindole (DAPI) staining and Annexin-V/FITC and PI staining methods. Briefly, CD44-enriched B16F10 cells seeded in two 6-well plates were exposed to ATRA or different NP formulations (5 μM of ATRA) for 48 h. After the treatment, one plate cells were washed with PBS, fixed by 4% poly formaldehyde solution and then incubated with DAPI (1 μg/mL) for 10 min to stain the nuclei. The nuclear morphology of cells was analyzed using a Life Technologies fluorescence microscope (Ex 358 nm, Em 461 nm). Cells were judged to be apoptotic based on nuclear morphology changes, including chromatin condensation, fragmentation and apoptotic body formation. The other plate cells were harvested and cell surface of phosphatidylserine in apoptotic cells was quantitatively estimated by using Annexin-V/FITC and PI apoptosis detection kit (KeyGEN BioTECH, China). The analysis of apoptotic cells was performed on a Beckman Coulter Navios flow cytometry.

### Evaluation of biodistribution property

An in situ lung metastasis model was constructed to evaluate the biodistribution property. In brief, mice were injected with 3 × 10^5^ B16F10 cells via the tail vein 3 weeks in advance. 1,1-Dioctadecyl-3,3,3,3-tetramethylindodicarbocyanine (DiD, KeyGEN BioTECH, China) was used to label the NP formulations. The mice were administered with NP formulations at an equivalent of 200 μg DiD/kg body weight for in vivo imaging. Then, the mice were anesthetized by injection with 3% isoflurane in oxygen, and the fluorescence signal of the mice were recorded (Ex = 640 nm; Em = 680 nm) using an IVIS^®^ Spectrum system (Caliper, Hopkington, MA). In addition, one other groups of mice were sacrificed at 8 h, and organs of interest were subjected to similar fluorescence tissue distribution measurements. The removed lungs were frozen in tissue freezing medium (Leica, Germany) and cut using a CM 1950 microtome (Leica, Germany) into sections, followed by 4′6-diamidino-2-phenylindole (DAPI) staining for fluorescence microscopy analysis.

### Side population analysis

The protocol was based on Naito et al. with slight modifications [46]. Briefly, CD44-enriched B16F10 cells seeded in 6-well plates were exposed to ATRA or NP formulations (5 μM of ATRA) for 48 h. The positive control cells were incubated with verapamil (100 μg/mL) for 20 min. After that, the cells were washed with PBS twice and harvested. Then, the cells were dispersed in fresh medium containing 2% FBS and 10 mM HEPES, and followed by incubating with Hoechst 33,342 (5 μg/mL final concentration) for 90 min at 37 °C with intermittent mixing. At the end of the incubation, cells were washed and suspended in cold PBS with 2% FBS and 10 mM HEPES. Five microlitre PI (50 μg/mL final concentration) was added 30 min ahead of flow cytometry analysis (BD biosciences, USA).

### In vivo tumorigenicity experiments

Basic procedures were previously described [47]. Briefly, CD44-enriched B16F10 cells seeded in 6-well plates were exposed to ATRA or different NP formulations (5 μM of ATRA) for 48 h. After the treatment, cells were harvested, resuspended in sterile PBS, and 1 × 10^3^ cells were injected subcutaneously into the flanks of mice. Tumor development was monitored after implantation. When tumor burden became obvious, the mice were sacrificed and tumors were harvested for analysis. The B16F10 tumor occurrence was calculated by the following equation.

3 $$ {\text{Tumor occurrence}}\%   = \frac{\text{the number of tumors}}{\text{the number of injections}} \times 1 0 0 $$

### In vivo antitumor experiments

A lung metastasis model was utilized for in vivo antitumor evaluation. Briefly, 5 × 10^5^ B16F10 cells were injected into C57BL/6 mice through the tail vein on day 0. The mice were randomly divided into 4 groups (PBS, ATRA, NPs/ATRA, and HA-eNPs/ATRA). ATRA-containing formulations were subsequently administered every 2 days at the dose of 5 mg/kg ATRA starting on day 14 and continuing until day 22. The body weight was monitored. At the desired time, mice were sacrificed and the tumor-bearing lungs were removed, weighted and fixed in 10% neutral-buffered formalin for H&E staining.

### Safety evaluation

Mice were administrated intravenously with ATRA-loaded NP formulations (5 mg/kg in ATRA) once every 2 days for 3 times. On day 7, the mice were sacrificed by anesthetizing with chloral hydrate. The visceral organs, including heart, liver, spleen, lung, kidney and brain, were removed, fixed in formalin, and embedded in paraffin. Serial 4-μm sections were prepared and stained with H&E for microscopic assessment.

### Statistics

Unless otherwise noted all quantitative data were presented as the mean ± SD from at least 3 independently experiments. Differences between two groups were analyzed by Student’s *t* test. Multiple comparisons of more than two groups, one-way ANOVA followed by post hoc t-test was performed. Statistical analysis was performed with SPSS/Win13.0 software (SPSS, Inc., Chicago, Illinois). Statistical significance was set at *P *< 0.05.

## Additional file

**Additional file 1.** **Fig. S1.** MALDI-TOF–MS spectrum of eBSA. **Fig. S2.** A working curve for quantification of HA content in HA-eNPs using Alcian blue assay. **Fig. S3** Difference on expression of CD44 between B16F10 and MCF-7 cells. **Fig. S4** In vitro antitumor effects of NP formulations on the MCF-7 cells. **Fig. S5.** Time-dependent intensity of fluorescence distribution of eNPs in mouse. **Fig. S6.** HA-eNPs/ATRA reduced the tumorigenicity of CD44-enriched B16F10 cells.

## Abbreviations

CSCs: cancer stem cells
TEM: transmission electronic microscopy
ATRA: all-trans-retinoic acid
HA: hyaluronic acid
EE: encapsulation efficiency
NPs: nanoparticles
HA-eNPs: nanoparticles system based on cationic albumin functionalized with HA
