# Supplementary material for: Specific cancer stem cell-therapy by albumin nanoparticles functionalized with CD44-mediated targeting
Source: J Nanobiotechnology. 2018 Dec 1;16:99. doi: 10.1186/s12951-018-0424-4 (PMC6271611; doi:10.1186/s12951-018-0424-4)
Supplement: Supplementary file 1 — Additional file 1. Fig. S1. MALDI-TOF–MS spectrum of eBSA. Fig. S2. A working curve for quantification of HA content in HA-eNPs using Alcian blue assay. Fig. S3 Difference on expression of CD44 between B16F10 and MCF-7 cells. Fig. S4 In vitro antitumor effects of NP formulations on the MCF-7 cells. Fig. S5. Time-dependent intensity of fluorescence distribution of eNPs in mouse. Fig. S6. HA-eNPs/ATRA reduced the tumorigenicity of CD44-enriched B16F10 cells. [file 12951_2018_424_MOESM1_ESM.docx]

**Additional file 1**

**Title:**

**Specific cancer stem cell-therapy by albumin nanoparticles functionalized with CD44-mediated targeting**

*Yuanyuan Li^#^, Sanjun Shi^#^, Yue, Ming, Linli Wang, Chenwen Li, Minghe Luo, Ziwei Li, Bin Li, Jianhong Chen^*^*

Department of Pharmacy, Third Affiliated Hospital & Research Institute of Surgery of Army Medical University, Chongqing 400042, PR China

^#^ These authors contributed equally to this work

^*^ Corresponding author: Jianhong Chen

Department of Pharmacy, Third Affiliated Hospital & Research Institute of Surgery of Army Medical University, 10# Changjiangzhilu, Chongqing 400042, PR China

E-mail: chenjh-110@263.net

Tel: 86-23-68757091

Fax: 86-23-68898867

**1 Characterization of ethylenediamine bond BSA (eBSA)**

The synthesized eBSA was analyzed by mass spectrum at room temperature using a Shimadzu 7090. The quantity of ethylenediamine groups bonded with each BSA molecule (MW = 66430 Da) was calculated by the following equation.

$$\text{T}\text{he number of }\text{ethylenediamine groups}\text{ }\text{bond with}\text{ }\text{each}\text{ BSA molecule}\text{ }$$

$$\text{= }\frac{\text{ molecular weight of eBSA (MW}\text{eBSA}\text{) }\text{ }\text{- }\text{MW}\text{BSA}}{\text{MW}\text{ethylenediamine}\text{ }\text{ - }\text{MW}\text{H2O}}\text{ }$$

…………………………… (Equation S1)

As shown in Fig. S1, the MW of eBSA was ~69614 Da. We can know there were on average 76 ethylenediamine groups bond with each BSA molecule.


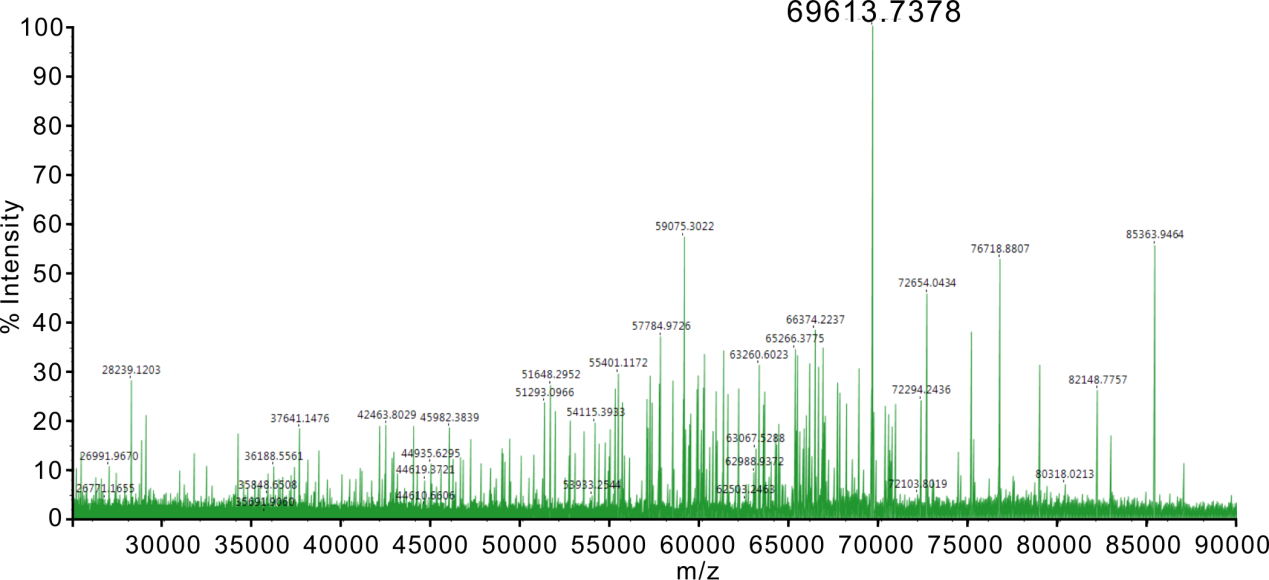


Fig. S1 MALDI-TOF-MS spectrum of eBSA.

**2 Determination of the content of HA on the surface of HA-eNPs.**

HA located on the surface of HA-eNPs was determined continually by a spectrophotometric method [1]. Briefly, 1 mL HA-eNPs solution was centrifuged at 12,000 rpm for 90 min. The supernatant (0.8 mL) was added into 1.2 mL alcian blue 8GX (Sangon, Shanghai, China) solution (0.14%, w/v) containing 0.5 M CH_3_COONa. Then, the mixed solution was shaked and followed by maintaining at 25 °C for 15 min. The absorbance of the solution was determined at 480 nm by a UV-vis spectrophotometer (Persee, Beijing, China). Absorption background from the control samples consisting of alcian blue 8GX alone was subtracted from the test samples. The amount of HA in the supernatant was derived from a working curve based on a series of standards at HA concentrations of 25-200 μg/mL. The content of HA located on the surface of the final particles was calculated by subtraction of the amount of HA in the supernatant from the total amount of HA. The density of HA on the surface of HA-eNPs was calculated by the following equation.

$$\text{HA density (}\text{}\text{g/mg}\text{) = }\frac{\text{amount of HA on the surface of the nanoparticles}}{\text{weight of the drug loaded HA-eNPs}}$$

…………………………… (Equation S2)


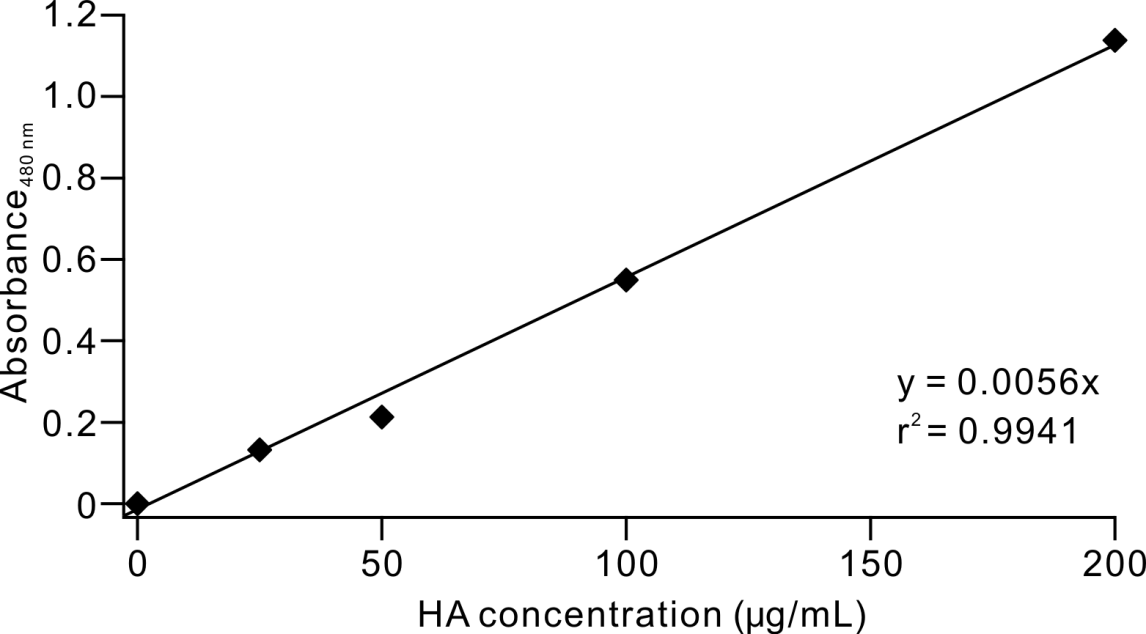


Fig. S2 A working curve for quantification of HA content in HA-eNPs using Alcian blue assay. Results are shown as the means ± SD (*n* = 3).

**3 Evaluation of CD44 expressions on B16F10 and MCF-7 cells by immunofluorescence assay**

For immunofluorescence, the untreated B16F10 and MCF-7 cells were seeded in a 6-well culture plate 1 day ahead. Then, the cells were fixed in 4% poly formaldehyde for 10 min at room temperature and washed three times with cold PBS, and blocked in PBST buffer containing 1% BSA and 22.52 mg/mL glycin for 30 min at room temperature. Cells were incubated overnight at 4 °C with rabbit monoclonal anti-CD44 (Abcam, ab189524) in blocking buffer. After that, cells were washed three times with cold PBS, incubated with anti-rabbit Alexa 488 (Abcam, ab150077) in blocking buffer for 1 h at room temperature, washed with PBS. Finally, cells were stained with DAPI for 5 min at room temperature, washed three times with PBS, and analyzed by a fluorescent microscopy.

As shown in Fig. S3, B16F10 cells showed strong green fluorescence signals especially on the cell membrane area, indicating high expression of CD44 on the cell surface. However, MCF-7 cells showed very weak fluorescence green signals in the whole cells, indicating low expression of CD44.


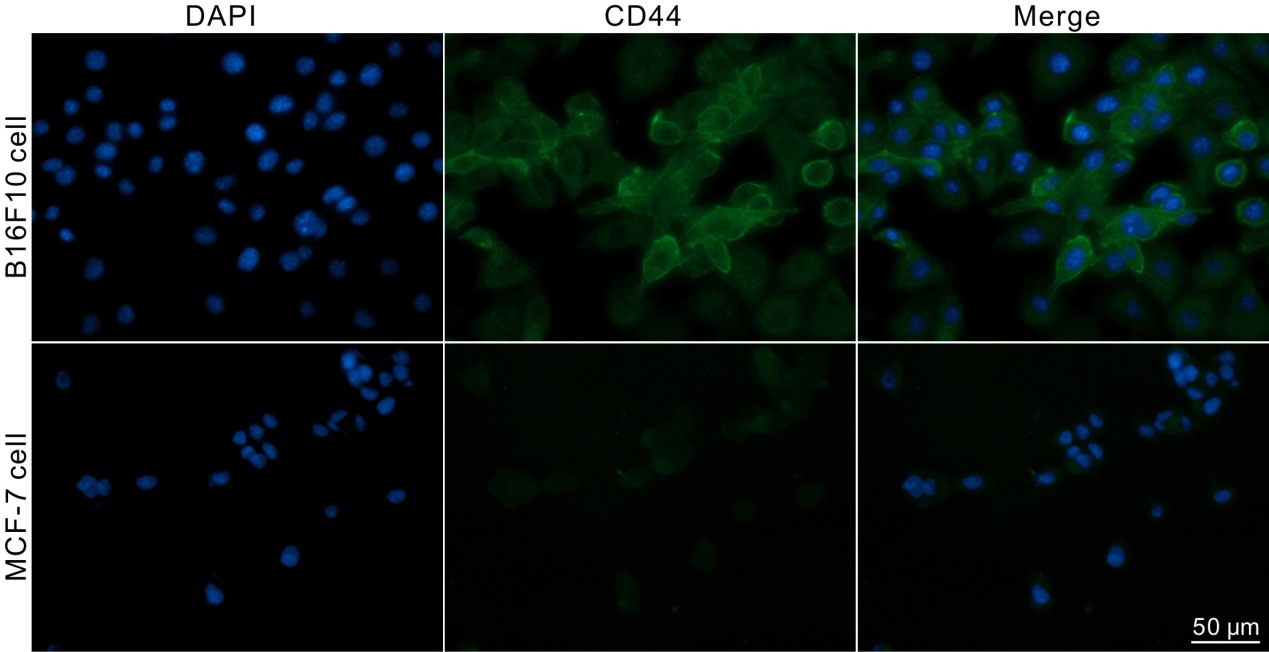


Fig. S3 Difference on expression of CD44 between B16F10 and MCF-7 cells. Blue fluorescence from DAPI-stained nucleus and green fluorescence from Alexa 488-labeled CD44 observed by fluorescence microscopy, as well as the merged image, are shown.

**4 Cytotoxicity evaluation of HA-eNPs/ATRA on CD44 low-expressing MCF-7**

To investigate the significance of HA modification on the nanoparticles, evaluation of cytotoxicity of HA-eNPs on the CD44 low-expressing MCF-7 cells was carried out. Briefly, MCF-7 cells were seeded in 96-well culture plates and allowed to attach. On the next day, the cells were exposed to ATRA or NP formulations for 48h at a series of ATRA concentrations and followed by addition of 20 μL MTT (5 mg/mL) to each well. The cells were continually incubated in total darkness for 4h. Then, the supernatant was replaced by 150 μL DMSO in order to ensure the formazan crystals dissolved. Cell viabilities were determined by measuring the absorbance at 570 nm using a Wellscan MK3 microplate reader (Thermo, USA).

As shown in Fig. S4, MCF-7 cell viabilities of all treated groups decreased with the increasing ATRA concentrations. The cytotoxicity of ATRA was slightly enhanced comparing with the free drug after encapsulated in NPs. However, HA modification on the surface of the NPs only reduced IC_50_ of ATRA less than 3 times (from 11.31 μM to 4.41 μM), which was partially resulted by the interaction of negative charged MCF-7 cells and positive charged HA-eNPs. On B16F10 cells, HA-eNPs greatly reduced IC_50_ of ATRA more than 7 times (from 3.6 μM to 0.49 μM, see Fig. 5b). These results told us that HA-eNPs could be selectively uptake by the CD44-enriched cancer cells.


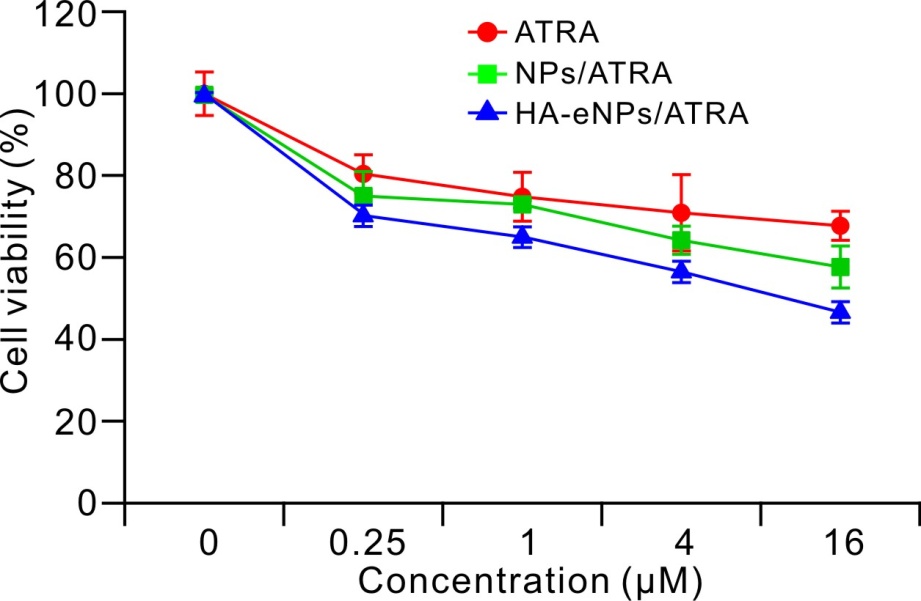


Fig. S4 *In vitro* cell viability assay of NP formulations on the MCF-7 cells. MTT viability assay for MCF-7 cells after treatment with various ATRA formulations. Results are shown as the means ± SD (*n* = 3).

**5 Biodistribution of eNPs/ATRA in lung metastasis tumor-bearing mice**

To verify whether HA modification facilitated cancer cell targeted delivery of ATRA, *in vivo* biodistribution of eNPs was performed. DiD was incorporated into eNPs as the fluorescence signals. DiD signals was monitored after introvenous administration of DiD-labeled eNPs into lung metastasis tumor-bearing mice (see details in the main text). Fig. S5 showed real-time images of eNPs in the tumor-bearing mice, in which the whole bodies were monitored over the course of 24 hours. Considerable fluorescence was detected and gradually decreased in the whole body over 24 hours, resulting from circulation of eNPs in the bloodstream. Interestingly, no increased fluorescent signal was observed in tumor-bearing lung, indicating that HA modification was necessary for cancer cell targeting.


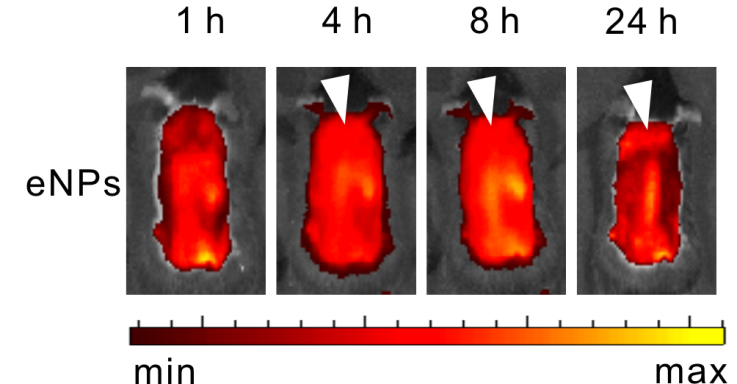


Fig. S5 Time-dependent intensity of fluorescence distribution of eNPs in mice (arrows indicate the position of tumor-bearing lungs).

**6 In vivo tumorigenicity experiments**

CD44-enriched B16F10 cells seeded in two 6-well plates were exposed to ATRA or different NP formulations (5 μM of ATRA) for 48 h. After the treatment, cells were harvested, resuspended in sterile PBS, and 1 × 10^3^ cells of different groups were injected subcutaneously into four flanks of the same mouse. Tumor development was monitored after implantation. After 24 days mice were sacrificed and tumors were harvested for analysis.


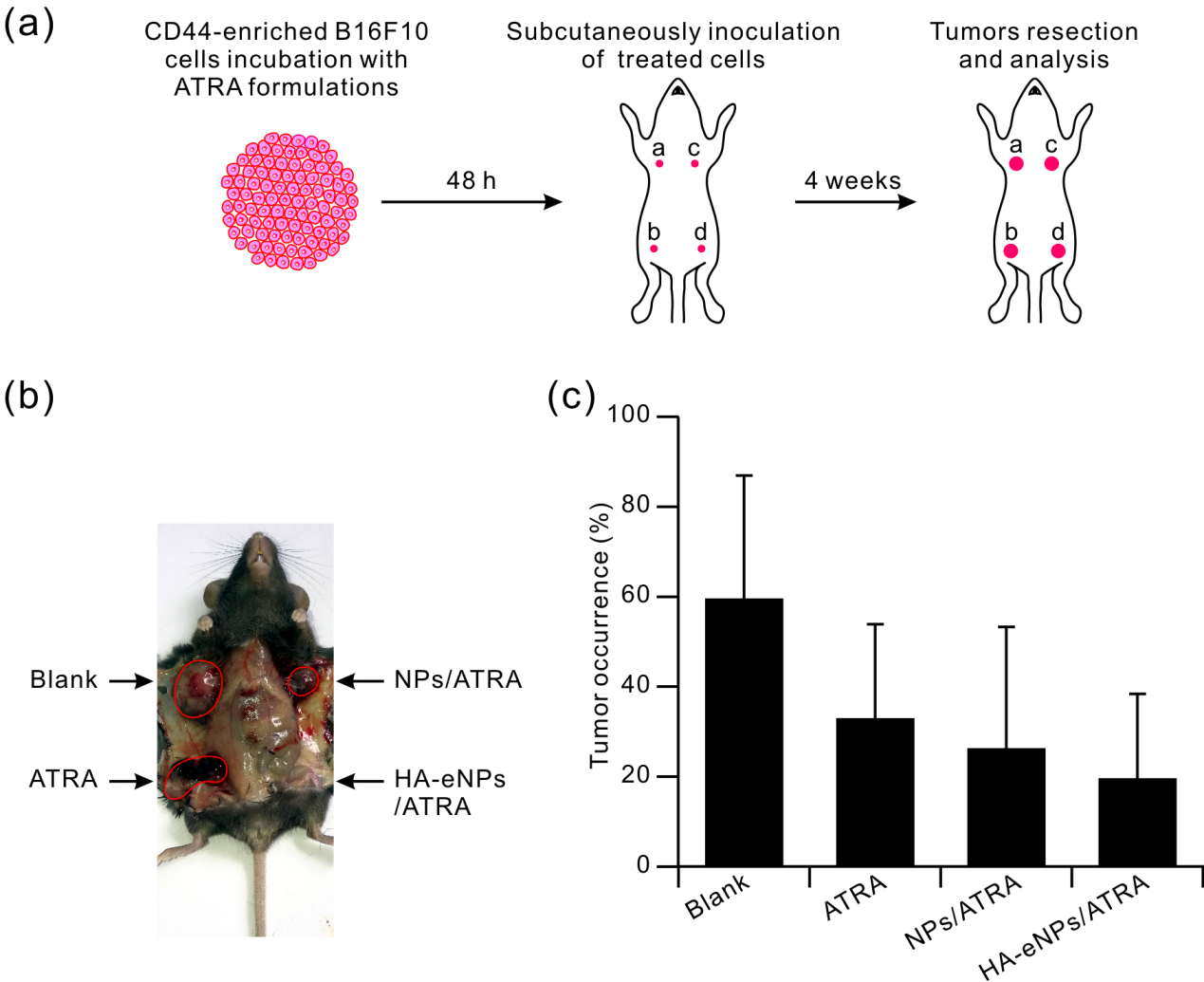


Fig. S6 HA-eNPs/ATRA reduced the tumorigenicity of CD44-enriched B16F10 cells. (a) A diagram of the tumorigenicity assessment. In the experiment, four flanks of each mouse were injected with the different treated or untreated B16F10 cells. (b) Representative photo of tumors 24 days after subcutaneous implantations of ATRA formulation treated and untreated B16F10 cells, arrows indicating the tumor nodules (c) Tumor occurrences evaluated 24 day after implantation. Results are shown as the means ± SD (*n* = 15).

Reference

1. Liu J, Zhou X.Y., Zhang H.Y., Zhang T., A simple spectrophotometric method for hyaluronic acid assay. Journal of Food Science and Biotechnology, 1995(1): 43-48.
